# The Role of Intercellular Adhesion Molecule-1 in the Pathogenesis of Psychiatric Disorders

**DOI:** 10.3389/fphar.2019.01251

**Published:** 2019-11-22

**Authors:** Norbert Müller

**Affiliations:** Department of Psychiatry and Psychotherapy, Ludwig-Maximilians-Universität Munich, Munich, Germany

**Keywords:** intercellular adhesion molecule-1, adhesion molecule, schizophrenia, depression, bipolar disorder, immunity, psychoimmunology

## Abstract

Intercellular adhesion molecule-1 (ICAM-1) is a transmembrane glycoprotein that is overexpressed in many pathological states. Although, like many other immune molecules, ICAM-1 plays only a limited role in the abundant concert of the immune response, it may be more important than we realize. In the central nervous system (CNS), ICAM-1 is expressed in microglial cells and astrocytes and in endothelial cells in the white and gray matter of the human forebrain. It is of particular interest in psychiatric disorders for two reasons: It has a key function for the blood–brain barrier, which plays an important role in the biology of psychiatric disorders, and it is a marker for inflammation. Although the blood level of soluble ICAM-1 (sICAM-1) might be lower in acute unmedicated schizophrenia, it has been reported to be increased in many other psychiatric conditions, such as major depression, bipolar disorder, and dementia. In bipolar disorder, high sICAM levels were found during both the depressed and the manic states and also during the euthymic phase (the free interval), possibly indicating that sICAM is a trait marker. High sICAM-1 blood levels have also been found in depression comorbid to a somatic disease state. Interestingly, sICAM-1 levels also increase during aging. Some studies investigated sICAM-1 levels in the cerebrospinal fluid of psychiatric disorders and ICAM-1 expression in postmortem CNS tissue of psychiatric patients and found that the overall duration and duration of the chronic phase of the psychiatric disorder seem to play a role in both. Moreover, confounders, such as antipsychotic and antidepressive medication, have to be considered. sICAM-1 levels seem to be associated with hypopermeability or hyperpermeability of the blood-brain barrier and thus to influence the communication between the CNS immune system, represented by glia cells, and the peripheral immune system. The balance between the influx and efflux of immune molecules into and out of the CNS may be one of the pinpoints in psychiatric disorders, in particular in the chronic phase, e.g., in schizophrenia. This aspect, however, needs further intense research, in particular to enable researchers to develop therapeutic principles based on an immune/inflammatory approach.

## Introduction

Psychotropic drugs such as antidepressants and antipsychotics act on serotonergic, noradrenergic, dopaminergic, and glutamatergic neurotransmission and are effective and well tolerated in the majority of patients. However, around one third of patients with depression are resistant to treatment and even fewer recover. In patients with schizophrenia, the effects of antipsychotics are often unsatisfactory because these drugs cannot prevent the chronic phase. These examples indicate that pathological mechanisms beyond those involving neurotransmitters may play an important role in psychiatric disorders and that research focused on neurotransmitters has reached its limits.

One important argument for the necessity of widening the view on the role of inflammation and the immune system in psychiatric disorders is that no pathogenetic cause has been found for the known dysfunction of monoaminergic neurotransmission, despite intense genetic and biochemical research over the last 40 years. Inflammation and immune dysfunction have direct and indirect influences on neurotransmission and thus may at least partly explain the biological dysfunctions in psychiatric disorders. For example, an activation of the inflammatory response system is well documented in schizophrenia and major depressive disorder (MDD) (Maes et al., 1992; Müller et al., 1993; Maes, 1994; Rothermundt et al., 2001; Myint et al., 2005; Müller and Bechter, 2013). Two meta-analyses on the role of cytokines clearly revealed similar changes in pro- and anti-inflammatory cytokines in various psychiatric disorders, such as MDD, schizophrenia, and bipolar disorder, in both the blood and the cerebrospinal fluid (CSF) (Goldsmith et al., 2016; Wang and Miller, 2018), although they also showed differences between disorders in the levels of the inflammatory markers C-reactive protein, interleukin (IL)-1, and tumor necrosis factor-α (Goldsmith et al., 2016; Wang and Miller, 2018). Other examples are that levels of the pro-inflammatory cytokine IL-6 during childhood predict the risk for later psychosis or depression (Khandaker et al., 2014) and that inflammatory or autoimmune diseases predict the risk for later schizophrenia or mood disorders (Benros et al., 2011; Benros et al., 2013).

In general, the inflammatory response system appears to be activated in psychiatric disorders, but the levels of the different markers vary across studies. The immune system is a highly divergent and differentiated system composed of numerous interconnected molecules. Therefore, at first it may seem unwarranted to discuss the role of just one molecule in psychiatric disorders. Nevertheless, this review will take a closer look at intercellular adhesion molecule-1 (ICAM-1), one of five intercellular adhesion molecules, because it is a well-characterized molecule that has important functions in the process of inflammation and has been studied in various psychiatric disorders as a representative of a pro-inflammatory immune response. It is of special interest because studies indicate that it is differentially expressed in different psychiatric disorders. This review will focus on the role of ICAM-1 in schizophrenia, depression, bipolar disorder, and dementia. It will not discuss ICAM-1 in anxiety disorders because research on immune parameters in this disorder is rare and no studies have been published on ICAM-1 in anxiety disorders.

## Function of ICAM-1 and Soluble ICAM-1

ICAM-1 is an immunoglobulin (Ig)-like transmembrane glycoprotein that is overexpressed on the endothelial lumen in many pathological states (Springer, 1990; Muro, 2007; Lawson and Wolf, 2009). It is ∼100 kDa in size and belongs to the immunoglobulin supergene family. The membrane-bound form of ICAM-1 serves as a counter receptor for the β_2_-integrins, CD11a/CD18 (LFA-1) and CD11b/CD18 (Mac-1), found on leukocytes. Interactions with membrane-bound ICAM-1 facilitate leukocyte transmigration across the endothelium of many cell types. A soluble form of the molecule, soluble intercellular adhesion molecule-1 (sICAM-1), is found in serum and other body fluids, including CSF (Schwarz et al., 1998). sICAM-1 may be generated by proteolytic cleavage and/or alternative splicing of mICAM-1 messenger RNA (Ramos et al., 2014). Like membrane-bound ICAM-1, sICAM-1 interacts with LFA-1/Mac-1 to compete with leukocyte binding to membrane-bound ICAM-1 (Tsakadze et al., 2006) and to stimulate leukocytes (Schmal et al., 1998). Besides endothelial cells, membrane-bound ICAM-1 is primarily expressed not only in microglia cells but also in astrocytes of the central nervous system (CNS), e.g., in the white and gray matter of the human forebrain (Lee and Benveniste, 1999). ICAM-1 expression in the CNS is often associated with glial fibrillary acidic protein (GFAP)-immunoreactive astrocytes (Miguel-Hidalgo et al., 2007); i.e., there is a co-localization of ICAM-1 and GFAP. ICAM-1 is of interest in psychiatric disorders for two reasons: 1) It has a key function in the blood–brain barrier (BBB), which plays an important role in the biology of psychiatric disorders by regulating the movement of molecules from the peripheral body, in particular components of the immune system, into and out of the CNS (see 2.1), and 2) it is a marker for inflammation.

Soluble ICAM-1, a circulating form of ICAM-1, arises from alternative splicing and proteolytic cleavage of membrane-bound ICAM-1 (Ramos et al., 2014). An interesting biological process associated with ICAM-1 overexpression during inflammation and its engagement by leukocytes is that of endothelial release of the ectodomain of ICAM-1, which can then circulate as sICAM-1 (Witkowska and Borawska, 2004; Lawson and Wolf, 2009). The literature describes sICAM-1 as an inflammatory marker and regulator that can promote the inflammatory response (Witkowska and Borawska, 2004; Lawson and Wolf, 2009). Serum levels of sICAM-1 appear to be low in healthy individuals, but they increase in many pathologies and are associated with disease progression and severity, for example, in cancer, cardiovascular disease, immune syndromes, and maladies involving chronic inflammation (Witkowska and Borawska, 2004; Lawson and Wolf, 2009). This association is not merely circumstantial, and sICAM-1 appears to be functionally involved in these diseases (Witkowska and Borawska, 2004). *In vitro* studies have shown a direct correlation between levels of shedded sICAM-1 and cell-surface ICAM-1, suggesting that increased levels of sICAM-1 in the CSF—as in the blood—are indicative of upregulation of surface-bound ICAM-1 in the brain (Leeuwenberg et al., 1992).

When discussing the role of ICAM-1 in psychiatric disorders, it must be noted that increased expression and shedding of ICAM-1 is observed in a wide range of diseases, only a few of which are neuropsychiatric. Inflammation, however, is a general condition of the human body, and the mechanisms of inflammation in psychiatric conditions parallel those of inflammatory disorders in non-psychiatric ones. A specific, important aspect of ICAM-1 in psychiatric disorders might be its role at the BBB.

### ICAM-1 and the BBB

In healthy states, there is an ongoing but limited communication between the CNS and peripheral immune system. In some CNS diseases, however, a disturbance or breakdown of the BBB leads to an uncontrolled invasion of components of the peripheral immune system into the CNS, where they are primed, e.g., by microglial cells; after re-entering the periphery, they act to activate or inhibit the peripheral immune system. Pro-inflammatory molecules lead to an activation of brain-derived cells, including microglia and astrocytes; these two cell types almost completely surround the capillary endothelial cells that make up the BBB and modulate the barrier *via* effects on these endothelial cells (Benveniste, 1992). During acute inflammation, immune cells invade the CNS parenchyma through the disturbed BBB, i.e., through the endothelium of the small vessels and the tight junctions of astrocytes around the vessels. Activation of endothelial cells and increased expression of ICAM-1 result in breakdown of the BBB and increase leukocyte recruitment, adhesion, and infiltration. This immune cell invasion is mediated by cytokines, chemokines, adhesion molecules, and other mediators of inflammation, and ICAM-1 is a key molecule in this process. The physiological purpose of this process is to identify and eliminate antigens in the CNS. For example, infections with a virus that does not activate glial cells have a considerably more unfavorable course than do infections with a virus that does stimulate an immune response that involves astrocytes (Lewandowski et al., 1994).

Abnormalities in the CSF that indicate a disturbance of the BBB are described in at least 20% to 30% of psychiatric patients. One study found that the BBB was disturbed in 27% of patients with schizophrenia, for example, and IgG was produced intrathecally in 15% of these patients (IgG was measured by the relatively “rough” method of analyzing albumin and IgG in the CSF) (Müller and Ackenheil, 1995). Interestingly, the amount of IgG in the CSF correlated significantly with the psychopathology of schizophrenia, in particular with negative symptoms. In MDD, pathological changes in the CSF were observed in about 25% to 30% of patients (Hampel et al., 1995). Much more severe disturbances of the BBB are seen in acute, fulminant inflammatory processes, e.g., bacterial or viral meningitis or encephalitis. The signs of a mild inflammatory process in a range of mental disorders prompted Müller and Bechter to formulate the “mild encephalitis hypothesis” (Müller and Bechter, 2013). The significant correlation between psychopathology and IgG levels indicates close links between immune and disease processes, at least in schizophrenia.

## ICAM-1 in Psychiatric Disorders

As described above, sICAM-1 can be determined in body fluids, whereas ICAM-1 expression is analyzed in tissue, including CNS tissue. The results of sICAM-1 and ICAM-1 evaluations in different psychiatric disorders are described below.

### Schizophrenia

A study of the *peripheral blood* in schizophrenia showed lower sICAM-1 levels in the blood of unmedicated patients than in healthy controls (Schwarz et al., 2000). During antipsychotic treatment lasting about 12 weeks, the study found a trend toward an increase in sICAM-1 and detected a relationship between sICAM-1 blood levels and schizophrenic psychopathology: Negative symptoms correlated positively with sICAM-1 levels, indicating that higher levels of sICAM-1 are associated with a more severe schizophrenic negative syndrome (Schwarz et al., 2000). The view that sICAM-1 levels are influenced by antipsychotic medication is supported by a study on the ICAM-1 ligand leucocyte function antigen-1 (LFA-1), which found that LFA-1 expression on leucocytes increased significantly during antipsychotic therapy (Müller et al., 1999). A recent study assessed sICAM-1 (and additional inflammatory adhesion molecules) in the plasma of 78 patients with schizophrenia or schizoaffective disorder and 73 healthy controls (Cai et al., 2018); interestingly, the authors found that sICAM-1 was significantly elevated in schizophrenia. However, the paper gives no information on antipsychotic treatment, so that a role of medication in the increase in sICAM-1 levels cannot be excluded. Another study described an association of sICAM-1 plasma levels with the stage of schizophrenia: In the early stage of schizophrenia, levels of sICAM-1 did not differ from those in healthy controls, but in the late stage of the disease, levels were higher than those in controls (Stefanovic et al., 2016). Of interest is that there was a significant relationship between sICAM-1 levels (but not levels of vascular cell adhesion molecule, VCAM-1, a cell adhesion molecule expressed on blood vessels) with clinical features; i.e., in the early stage of schizophrenia, sICAM-1 was related to the severity and type of current psychopathology, whereas in the late stage, it was associated with the progression of the disorder (Stefanovic et al., 2016). In the early stage of schizophrenia, patients with higher acute sICAM-1 levels had a less favorable treatment response. Similarly, in the late stage of schizophrenia, patients with a longer duration of untreated psychosis and shorter lifetime exposure to treatment had higher sICAM-1 levels compared with the early episode. In addition, higher daily chlorpromazine-equivalent doses applied at the initiation of the psychotic episode and maintained to the remission phase resulted in significantly lower sICAM-1 levels in patients in the late stage of the disease (Stefanovic et al., 2016). These findings are only partly in line with those of Schwarz and colleagues reported above (Schwarz et al., 2000). Many studies have described an increase of pro-inflammatory markers in patients with a longer duration of disease and longer treatment period. A possible explanation why Stefanovic and colleagues did not find decreased sICAM-1 in unmedicated patients with schizophrenia may be that the patients in their study were in different clinical and psychopathological states.

Levels of **sICAM-1 in the CSF** better reflect the environmental condition of the CNS than do blood levels. However, valid CSF studies are lacking. A small study of sICAM-1 levels in the CSF of schizophrenia patients found significantly lower levels than those in healthy controls and in patients with a non-inflammatory neurological disorder (Schwarz MJ, unpublished results). Another study showed that sICAM-1 levels in the CSF are related to a disturbance of the BBB (Schwarz et al., 1998), a finding that supports the role of ICAM-1 in the BBB.

A recent study in schizophrenia patients estimated **ICAM-1 expression in the brain**. ICAM-1 mRNA expression in the prefrontal cortex was compared in a “high inflammation” schizophrenia subgroup—defined *a priori* acording to a cluster of cortical pro-inflammatory cytokine mRNAs in the patients—a low inflammation subgroup, and healthy controls. The study found that mRNA expression was higher in the “high inflammation” subgroup than in the “low inflammation” subgroup and controls (Cai et al., 2018). The role of antipsychotic medication and other confounding factors, however, needs to be evaluated in further studies.

An immunogenetic influence of the ICAM **G241A** or **A469G polymorphisms** in schizophrenia could not be detected, and no difference in sICAM-1 levels was found (Riedel et al., 2003). In healthy control persons carrying the polymorphic A allele (G241A), however, markedly lower sICAM-1 serum levels were found than in carriers of the homozygous GG wild type (G241G) (p < 0.004) (Kronig et al., 2005).

An overview of studies on ICAM-1 in schizophrenia is provided in **Table 1**.

**Table 1:** ICAM-1 in schizophrenia: overview of findings.

Author	Source	n	Method	Result
Cai et al., 2018	Prefrontal cortex tissue	37 schizophrenia/schizoaffective psychosis no information on medication vs 37 healthy controls	PCR	Higher ICAM-1 mRNA expression (p < 0.05)
	Plasma	78 schizophrenia/schizoaffective (medicated) vs 73 healthy controls	Luminex	Significantly higher sICAM-1 levels (p < 0.01)
Müller et al., 1999	Serum CSF	45 schizophrenia patients (unmedicated) 22 schizophrenia patients (medicated) 32 schizophrenia patients (CSF)	FACS	ICAM-1 ligand leucocyte function antigen-1 expression on leucocytes increased significantly during antipsychotic therapy
Schwarz et al., 1998	CSF	40 schizophrenia patients (medicated)	ELISA	Significant relationship of SICAM-1 to blood–CSF barrier
Schwarz et al., 2000	Serum CSF	36 unmedicated schizophrenia 36 medicated schizophrenia 38 healthy controls 18 schizophrenia (CSF)	ELISA	Trend toward significantly lower sICAM-1 levels in unmedicated and medicated schizophrenia patients; increase of sICAM-1 during treatment
		ICAM-1 in schizophrenia: overview on findings		Significant positive correlation of sICAM-1 with negative symptoms and duration of disease
Stefanovic et al., 2016	Serum	80 schizophrenia (medicated) (40 early stage, 40 late stage) 80 matched healthy controls	ELISA	sICAM-1 levels normal in early-stage schizophrenia, higher in late-stage schizophrenia. Related to disease severity and cognitive and excitement symptoms in early stage. Related to disease duration in late stage

ICAM-1, intercellular adhesion molecule-1; CSF, cerebrospinal fluid; sICAM-1, soluble ICAM-1; FACS, fluorescence-activated cell sorting.

### MDD

In the past decade, a huge number of studies have been published on pro- and anti-inflammatory markers, and a role for inflammation in at least a subgroup of depressed patients is well established. Interestingly, the role of ICAM-1 has been poorly evaluated, in particular in MDD patients without co-morbidities. Because accompanying depressive states often occur in somatic disorders and especially in inflammatory disorders—one of the starting points for research on the role of inflammation in depression—the function of ICAM-1 has often been studied in depressed states comorbid to somatic disorders. Moreover, because aging is associated with an increase in the pro-inflammatory immune state in healthy people (immunosenescence) (Fulop et al., 2017), research has focused more on the role of ICAM-1 in elderly people.

Various research groups have studied *blood levels of sICAM-1*, as well as of other adhesion molecules, in late-life depression. A recently published meta-analysis of data from 43,600 participants aged >40 years, which included 9,203 people with depression, found an association between higher sICAM-1 levels and depression (van Agtmaal et al., 2017). Another study examined the relationship between sICAM-1 and depression and found significantly higher sICAM-1 levels in MDD patients after a 3-day wash-out of antidepressants than in age- and sex-matched healthy controls (Baghai et al., 2018). The authors took into account a medication and treatment effect because they described a trend toward an increase in sICAM-1 levels during treatment before discharge. Nevertheless, a 3-day wash-out may be too short to exclude an effect of medication on sICAM-1. So far, six different meta-analyses have presented pooled odd ratios. These analyses showed a significant association between high levels of sICAM-1 (and some other adhesion molecules) and symptoms of depression in different samples of elderly people (Lesperance et al., 2004; Dimopoulos et al., 2006; Thomas et al., 2007; van Sloten et al., 2014; Tchalla et al., 2015; van Dooren et al., 2016). Most of the participants were not diagnosed with depression, but depression scores were higher in people with higher sICAM-1 levels (odds ratio 1.58, 95% confidence interval 1.28–1.96). Functionally, the authors interpreted the results as showing that generalized microvascular dysfunction is associated with depression. Multiple markers of microvascular dysfunction, including endothelial plasma markers and markers of cerebral small vessel disease, were cross-sectionally associated with a higher level of depressive symptoms and depressive disorder. These meta-analyses support the view that higher sICAM levels are associated with more severe psychopathology, here with symptoms of depression.

*sICAM-1 blood levels* in depressive disorder comorbid with a somatic disease, e.g., diabetes, have been studied in different conditions. An often-studied psychoneuroimmunological model is treatment with interferon-alpha. In interferon-alpha–treated patients, an increase in sICAM-1 levels was observed in parallel to an increase in depression scores, and significantly higher sICAM levels were found in more severely depressed patients (Schaefer et al., 2004). sICAM-1 blood levels were also examined in a study that aimed to investigate whether biomarkers of inflammation were associated with symptoms of depression in individuals with recently diagnosed diabetes (Herder et al., 2017). The authors were also interested to investigate whether such associations may differ by diabetes type. In contrast to the findings in people with type 2 diabetes, in people with type 1 diabetes, serum levels of sICAM-1 were positively associated with the score on the German version of the Center for Epidemiological Studies Depression Scale (Allgemeine Depressionsskala, Langversion).

*CSF levels of sICAM-1* (and other pro-inflammatory markers) were assessed in patients after CNS trauma and found to predict the risk for developing a posttraumatic depressive syndrome (Juengst et al., 2015), providing further evidence of the close relationship between sICAM-1 levels and the psychopathology of depression.

The *CNS expression of sICAM-1* may best reflect the relationship between the psychopathological state of depression and the role of ICAM-1. One interesting study compared the expression of ICAM-1 in CNS tissue in patients with unipolar and bipolar depression, patients with schizophrenia, and healthy controls (Thomas et al., 2004). The study found significant increases in the expression of ICAM-1 (and additional CAMs) in the gray matter of the dorsolateral prefrontal cortex (DLPFC) in the unipolar depression group but no comparable differences between groups in the anterior cingulate cortex (ACC) or occipital cortex (Thomas et al., 2004). It also found a non-significant increase in ICAM-1 in the white matter of the DLPFC in the unipolar depression group (but no increase in the other areas of the brain or of VCAM-1 in any area). Paired tests showed the specificity for the DLPFC in the unipolar depression group only (Thomas et al., 2004). Another group of researchers estimated the expression of ICAM-1 in the choroid plexus (ChP), a highly vascularized tissue in the CNS that produces CSF, does not have a BBB and is an interface between the peripheral and central immune responses (Devorak et al., 2015). The authors found lower ICAM-1 expression in suicide victims with depression than in controls. Because ICAM-1 is expressed by choroid epithelial cells and is thought to support immune cell trafficking in the ChP, this finding might be an indication of decreased immune cell trafficking through the BBB in depression and suicide. ICAM-1 in the choroidal epithelium is also known to be upregulated in response to exposure to acute pro-inflammatory molecules *in vitro*. The observed downregulation of ICAM-1 expression in the ChP may represent a compensatory mechanism that functions to counteract (chronically) increased activation of pro-inflammatory signaling pathways elicited by chronically elevated cytokine levels, which are frequently observed in depressed patients. A decrease in immune cell trafficking through the BBB from the periphery into the CNS or vice versa could consequently limit central and/or peripheral pro-inflammatory signaling (Devorak et al., 2015). A converse interpretation of the finding might be that under certain psychopathological conditions the BBB shows hypopermeability and thus does not allow adequate molecular interchange between the CNS and peripheral immune system, so that immune activation in the CNS cannot be cleared.

### Bipolar Disorder

As mentioned in the above discussion of the role of ICAM-1 in depression, the majority of studies to date did not examine individuals with a diagnosis of depression, but with a depressed state of varying severity. Accordingly, they did not differentiate between different types of depression, e.g., recurrent depression or bipolar depression. However, some studies have evaluated individuals with bipolar disorder, during either a manic or a euthymic state.

One study found a weak positive correlation between sICAM-1 levels and the scores on the Yale Mania Rating Scale in patients in a manic state (Turan et al., 2014). In their study in patients with bipolar disorder (n = 83), another group of researchers described higher blood levels of sICAM-1 (and higher IL-6, but lower tumor necrosis factor-α and lower sVCAM) in both acute and remission phases of the disorder than in age-, sex-, and body-mass–matched healthy controls (n = 73) (Pantovic-Stefanovic et al., 2016). Similarly, another study found significantly higher blood levels of sICAM-1 in euthymic bipolar patients than in healthy controls (Reininghaus et al., 2016). This study did not find an association with smoking, sex, or the duration of disease, but patients in a progressive stage of the disease had higher levels of sICAM-1 than did those in an earlier stage. Higher sICAM-1 levels were also found in patients on atypical antipsychotics, whereas prophylactic treatment with lithium or antiepileptics showed no association with sICAM-1 levels (Reininghaus et al., 2016). This finding is in line with earlier results showing signs of an immune activation during the euthymic phase (or free interval) of bipolar patients (Müller et al., 1993). It also corresponds with an interesting finding in a sample of healthy people (without a psychiatric diagnosis) who were grouped according to their affective temperament: In a multiple linear regression model, sICAM-1 blood levels were not related to affective states per se but to the state severity of manic symptoms measured with the Yale Mania Rating Scale (Ivkovic et al., 2017).

*In CNS tissue*, increased expression of ICAM-1 was described in both the gray and white matter of the ACC in bipolar patients compared with controls (gray: *p* = .001; white: *p* < .001) and schizophrenia patients (gray: *p* = .016; white: *p* = .025), and modestly increased expression was described in the white matter of the ACC in bipolar patients compared with patients with unipolar depression (*p* = .049). No differences were found in the DLPFC (Thomas et al., 2004).

### Dementia

Aging is the main risk factor for Alzheimer’s disease (AD), and a dramatic increase in extravascular ICAM-1 (associated with GFAP-immunoreactive astrocytes) is seen in the orbitofrontal cortex in normal aging. This increase may contribute to an enhanced risk for CNS inflammatory processes during aging (Miguel-Hidalgo et al., 2007). Decades ago, ICAM-1 was found to be increased in age-related neurodegenerative diseases (Akiyama et al., 1993). Accordingly, high ICAM-1 levels can be expected in AD. Besides localizing to the brain vasculature, in AD ICAM-1 also localizes to amyloid plaques, the biological hallmark of the disease, forming extravascular aggregates (Verbeek et al., 1994). It has been shown that these ICAM-1 immunoreactive aggregates are mostly absent in non-demented control individuals, even in the presence of normal vascular ICAM-1 immunoreactivity (Lee and Benveniste, 1999). However, it is still unclear whether age-related changes in extravascular ICAM-1 immunoreactivity occur in the CNS of normal, non-demented individuals, even though inflammatory processes in the CNS are well known to increase with aging (Bodles and Barger, 2004) and increases observed in neurodegenerative disorders could be age related. In animal models of brain injury, levels of mRNA for ICAM-1 and inflammatory cytokines increase more in old than in young rats (Kyrkanides et al., 2001), in parallel with increased immunostaining of GFAP in astrocytes. Furthermore, in humans, there seems to be an age-related increase in astrocytic GFAP immunoreactivity in the cerebral cortex (Prolla and Mattson, 2001) that might be paralleled by changes in key inflammatory molecules, particularly ICAM-1. In fact, astrocytes express ICAM-1 immunoreactivity *in vitro* and *in vivo* after brain damage (Lee and Benveniste, 1999).

The direct association between ICAM-1 and neurodegeneration has not yet been intensely studied. An *analysis of plasma sICAM-1* in patients with AD or Lewy body dementia (LBD) revealed significantly higher sICAM-1 levels in both patient groups than in healthy controls (p < 0.001). *In the CSF*, however, only LBD patients showed higher sICAM-1 levels (and significantly lower sVCAM-1 levels) than AD patients and healthy controls (p < 0.001) (Nielsen et al., 2007). As expected, the study found a strong correlation between sICAM-1 levels and BBB permeability in the AD patients and controls, but not in the LBD patients. A further evaluation of the data revealed a significant correlation between CSF sICAM-1 and kynurenic acid, a metabolite of tryptophan/kynurenine metabolism that has N-methyl-d-aspartate antagonistic effects and is driven by pro-inflammatory cytokines. Interestingly, the levels of kynurenic acid significantly correlated with the AD biomarker phosphorylated tau, supporting the association between sICAM-1 CSF levels and neurodegeneration markers in AD (Wennstrom et al., 2014).

A large study in nearly 800 individuals showed that CSF levels of sICAM-1 (and other markers of neuroinflammation) were increased not only during the dementia stages of AD, but already during the preclinical and prodromal stages of the disease. High levels of sICAM-1 were associated with increased CSF levels of total tau and phosphorylated tau. These associations were found in both Aß-positive and Aß-negative individuals, although the association was stronger in the former. The results were similar for associations between phosphorylated tau and ICAM-1. High levels of sICAM-1 were also associated with cortical thinning (primarily in the precuneus and superior parietal regions) and with subsequent cognitive deterioration in patients without dementia, as measured with the Clinical Dementia Rating Scale Sum of Boxes. Finally, higher levels of CSF ICAM-1 increased the risk of developing AD dementia in patients without dementia (Janelidze et al., 2018). The authors concluded that neuroinflammation and cerebrovascular dysfunction are early events that occur at presymptomatic stages of AD and contribute to disease progression.

*In postmortem tissue* of demented patients, in AD an increased expression of ICAM-1 was detected in amyloid plaques and astrocytes around plaques (Akiyama et al., 1993). A recent study of 143 cases of LBD determined levels of ICAM-1 (and the anti-inflammatory molecule CD200) in postmortem CNS tissue, in particular in the temporal and cingulate cortex, and found that high levels of ICAM-1 expression correlated with the density of amyloid plaques and neurofibrillary tangles; moreover, there was a strong positive correlation between phosphorylated tau levels and ICAM-1 (Walker et al., 2017). In this study, ICAM-1 expression correlated more with the typical AD pathology (and phosphorylated tau as a general marker of neurodestruction) than with the typical LBD hallmarks.

## Discussion/Conclusion

In recent decades, psychoneuroimmunological research has come into the focus of biological psychiatry, and it is now widely accepted that mild inflammation plays a role in many different psychiatric disorders. The data indicate that ICAM-1 may be a key molecule in various psychiatric disorders. This view is supported by findings that ICAM-1 is related to clinical features of schizophrenia, depression, and bipolar disorder and to specific neurodegeneration markers in dementia. The results of studies on sICAM-1 are more consistent than those of studies on several other adhesion molecules, such as VCAM-1, ICAM-3, platelet endothelial cell adhesion molecule-1, and others, possibly because sICAM-1 is biologically more stable and easier to measure. ICAM-1 has more diverse functions than sICAM-1. On the one hand, it is a marker for inflammation in the periphery and CNS, and on the other, it reflects the permeability of the BBB and the communication between the CNS and peripheral immune system.

Decreased levels of sICAM-1, as partly described in schizophrenia, may result from dysfunctional neuroendocrine-immune communication, wherein an adequate immune response is not mounted or, alternatively, neuroinflammation is prolonged. A decrease in immune cell trafficking through the BBB from the periphery into the CNS or vice versa could consequently limit central or peripheral pro-inflammatory signaling or both (Devorak et al., 2015). Another interpretation of this finding might be that under certain psychopathological conditions the BBB is hypopermeable, which prevents adequate molecular interchanges between the CNS and peripheral immune systems and does not allow immune activation in the CNS to be cleared. Increased levels of sICAM-1 reflect an at least partial breakdown of the BBB, with the invasion of pro-inflammatory molecules into the CNS. This invasion is necessary to clear an infectious or inflammatory process in the CNS; however, once the process is complete, the inflammatory process must be downregulated; i.e., the number of anti-inflammatory molecules in the CNS and at the BBB must be increased. A disturbance of this regulatory process might be the key factor in diverse psychopathological states.

As a final point, one has to ask whether the increase in sICAM-1 levels and expression of ICAM on tissue reflect an unspecific process and why this increase in sICAM-1 is specifically associated with different disorders, including depression, schizophrenia, dementia, or bipolar disorder. The answer is provided by postmortem studies of CNS tissue, which reveal that different CNS regions are involved in the neuroinflammatory process in different psychopathological states, e.g., the DLPFC in depression and the ACC in bipolar disorder. *Ex vivo* neuroimaging, e.g., positron emission tomography, may be able to provide additional information in answer to these questions, but to date, no ligand for ICAM has been developed. The differences in the neuroinflammatory process between disorders may also explain why anti-inflammatory treatment has beneficial therapeutic effects in some psychiatric disorders, such as MDD and schizophrenia (Müller, 2015), and prophylactic effects in dementia (Müller et al., 2015).

## Author Contributions

NM, the sole author, was responsible for all aspects of the manuscript.

## Funding

The work was supported by the Foundation “Immunität und Seele.

## Conflict of Interest

The author declares that the research was conducted in the absence of any commercial or financial relationships that could be construed as a potential conflict of interest.
